# Major Plant-Based Compounds for the Prevention and Treatment of Melanoma—A Mini Review

**DOI:** 10.3390/biology14121772

**Published:** 2025-12-11

**Authors:** Isabella Kirshteyn, Megha Srivastav, Karen Grace, Victoria Cescato, Ajay Bommareddy

**Affiliations:** 1 College of Medicine, Florida State University, Tallahassee, FL 32304, USA; 2 Department of Biomedical Science, Charles E. Schmidt College of Medicine, Florida Atlantic University, 777 Glades Road, Boca Raton, FL 33431, USA

**Keywords:** melanoma, curcumin, sulforaphane, EGCG, resveratrol, quercetin, fucoxanthin, lycopene

## Abstract

Melanoma, particularly the malignant type owing to its aggressive metastatic potential, is one of the most severe cancers. When detected early, the cure rate of melanoma is very promising and has a higher 5-year survival rate. However, it becomes very difficult to treat when it has spread to deeper layers of the skin and to other parts of the body. Despite the reduced mortality, the incidence of melanoma is on the rise due to exposure to various environmental factors, particularly UV radiation without protection. Naturally occurring dietary agents have been studied for their antitumor and chemopreventive potential against the development of various cancers including melanoma. The current review is aimed at compiling developments in the past ten years surrounding their preclinical and clinical relevance in the treatment and prevention of malignant melanoma. Various cellular pathways modulated by these phytochemicals are also examined to provide a comprehensive overview of their mechanisms involved in reducing tumor burden. Specifically, the review focuses on the most consumed foods across the world that are rich in such phytochemicals including curcumin, sulforaphane, resveratrol, quercetin and epigallocatechin gallate (EGCG) and their potential against the development of melanoma.

## Simple Summary

Melanoma is the most aggressive type of skin cancer, and the prognosis for metastatic melanoma is poor. Despite the recent advancements in therapeutic options, the extension of life offered by these treatment strategies is only a matter of months due to the rapid development of resistance, which could lead to no response or a decreased response rate after the initial relief. The need for improved efficacy of current melanoma treatments calls for innovative strategies, such as the elucidation of combination therapies, continued discovery of novel therapeutic targets, and preclinical investigation of natural agents as adjuvant therapy. The antitumor potential and relatively low toxicities associated with the naturally occurring phytochemicals make them an attractive treatment option for metastatic melanoma. This review summarizes major plant-based compounds, such as curcumin, sulforaphane, epigallocatechin gallate, resveratrol, fucoxanthin, lycopene, and quercetin, that are most consumed through various foods and their role in the prevention and development of melanoma skin cancer.

## 1. Introduction

Melanoma, an aggressive and deadly skin cancer, develops from cutaneous melanocytes that are present in the basal layer of the epidermis. The incidence of melanoma has increased steadily over the past three decades compared to other cancer types and is one of the most common cancers diagnosed among young adults in the United States. It is estimated that 104,960 new cases of melanoma will be diagnosed and 8450 people are expected to die from this devastating disease in 2025 [1]. Established risk factors for the development of melanoma include features, like light-colored skin, hair, and eyes, and exposure to ultraviolet radiation. In particular, the genetic background of individuals, the extent of UV radiation exposure, and blistering sunburns early in life have been shown to play a causal role [2,3]. Five to fifteen percent of individuals with cutaneous melanoma have been identified to have a family history of melanoma, but a direct association of hereditary melanoma due to a transmitted genetic mutation is less common [4,5]. Cyclin-dependent kinase inhibitor 2A [*CDKN2A*] gene and cyclin-dependent kinase 4 (*CDK4*) gene germline mutations, among other less commonly known mutations, are frequently identified genetic abnormalities in familial melanomas [5,6]. Xeroderma pigmentosum [XP], an autosomal recessive disease is also associated with an increased risk of skin cancers, including melanoma.

The management of melanoma is complex and typically includes surgery, immunotherapy, targeted therapy, and radiotherapy [7]. Surgery is curative for the majority of patients who have cutaneous melanomas of low thickness [8]. However, rates of survival drop predominantly with increased tumor thickness due to the increased risk of metastasis [8]. This transition from a mostly benign disease to one with a more serious prognosis occurs as melanoma progresses through the radial and vertical growth phases. The prognosis for metastatic melanoma is grim: 5-year survival ranges from 12–28%, depending on the location of the metastasis [8]. Traditional cytotoxic therapy and immunomodulatory agents have failed to demonstrate significant efficacy, with fewer than 5% of patients having complete responses at 5 years [8]. Fortunately, the therapeutic landscape of unresectable melanoma has been transformed by the advances in targeted therapies and immune therapies. The new approaches have offered significant outcomes with improved survival when compared with the traditional strategies [4]. Yet, despite these advancements, the extension of life offered by these strategies is only a matter of months due to the rapid development of resistance, which could lead to no response or a decreased response rate after the initial response. The agents also target a fraction of the oncogenic signaling pathways that play a role in melanoma. Limitations regarding toxicity and patient safety have shifted interests toward the exploration of natural compounds for the treatment of melanoma.

Natural products have received increasing attention in recent years as novel cancer preventive and therapeutic agents. Preclinical studies employing naturally occurring phytochemicals, both alone and in combination with traditional cytotoxic and targeted therapies, have yielded promising results [8]. The relatively low toxicities of these substances make the adjuvant use of natural agents an attractive treatment option for metastatic melanoma. The need for improved efficacy of current melanoma treatments calls for innovative strategies, such as the elucidation of combination therapies, continued discovery of novel therapeutic targets, and preclinical investigation of natural agents as adjuvant therapy. This review aims to critically appraise the current knowledge on the most consumed foods across the world that are rich in such phytochemicals including curcumin, sulforaphane, resveratrol, quercetin, and epigallocatechin gallate (EGCG) and their potential against the development of melanoma. By focusing on the role of these natural products, we seek to enrich our current understanding of their involvement against the development of melanoma. The information compiled could serve as a guide for future directions and offer insights into the development of diverse therapeutic regimens for melanoma. In this review, we compiled information by performing a literature search using the keywords “melanoma” and “Curcumin, sulforaphane, resveratrol, quercetin, EGCG, Fucoxanthin and Lycopene” from 2015 until August 2025 using PubMed and Google scholar. A search of ClinicalTrials.gov using the keywords “resveratrol or other phytochemicals” and “melanoma” was performed, along with a broader search of ClinicalTrials.gov using the terms “resveratrol or other phytochemicals” and “cancer” for studies in other cancer models to summarize available clinical trial data. Similarly, a search on PubMed [pubmed.ncbi.nlm.nih.gov] with the same keywords and filters applied for Adaptive Clinical Trial, Clinical Study, and all phases of Clinical Trials ([I–IV]) was also performed to obtain information on clinical studies.

### 1.1. Quercetin

Quercetin, a polyphenolic flavonoid present in fruits, vegetables, and tea, has demonstrated antioxidant, anti-inflammatory, and anti-cancer activity in various cancers, including melanoma [2]. Quercetin contains a low-toxicity profile while demonstrating inhibitory effects on the migration and invasion of melanoma [9]. This section reviews current literature on quercetin and its antitumor potential, focusing on the molecular mechanisms, discoveries from in vivo and in vitro studies, synergistic interactions with other agents, and current evidence from clinical studies.

Numerous in vitro studies have demonstrated quercetin’s inhibitory effects on melanoma cell proliferation. Quercetin induces apoptosis and suppresses proliferation in a concentration-dependent manner, primarily through the induction of cell cycle arrest at the G1 phase. This effect is mediated by downregulating key G0/G1 regulatory proteins including cyclin D, cyclin E, CDK4, and CDK6, thereby disrupting cell cycle progression and cellular replication [10]. Furthermore, quercetin has been found to modulate melanogenesis, the biosynthetic pathway of melanin that depends on tyrosinase and tyrosinase-related proteins TRP-1 and TRP-2 [11]. In murine B16-F10 melanoma cells, quercetin inhibited tyrosinase activity, leading to reduced melanin synthesis and decreased cancer cell viability [11]. These findings highlight quercetin’s role in attenuating both melanoma cell growth and melanogenesis-related pathways.

Quercetin has also been shown to modulate key signaling pathways that are frequently dysregulated in melanoma. Specifically, it downregulates the PI3K/Akt and MAPK/ERK pathways, both of which are central to melanoma cell growth, proliferation, and survival [12]. Treatment with quercetin reduces the level of phosphorylated Akt and ERK, decreasing cell survival and increasing pro-apoptotic signaling. In addition, quercetin induces mitochondrial-dependent apoptosis by suppressing the anti-apoptotic protein Bcl-2 and activating caspase-3 and caspase-9 [12,13].

In vivo studies have investigated the tumor-suppressive potential of quercetin in murine models of melanoma. Treatment of mice xenografted with B16-F10 or A375 melanoma cells with quercetin resulted in significant inhibition of tumor growth. Quercetin treatment was associated with upregulated IFN-α and IFN-β, which exert antiproliferative effects on melanoma cells [14]. Further studies have revealed quercetin’s enhanced antitumor immunity by increasing the percentage of immune effector cells, including CD8+ T lymphocytes, CD4+ T lymphocytes, and M1 macrophages, promoting phagocytosis and contributing to an immunogenic tumor microenvironment [15].

Quercetin has demonstrated synergistic effects when used in combination with conventional chemotherapeutic agents and natural bioactive compounds. For example, quercetin complexed with cerium ions has been shown to form a thermodynamically favorable structure that can enhance its bioactivity [16]. When applied to A375 melanoma and MDA-MB-231 breast cancer cell lines pre-treated with photodynamic red-light therapy, the quercetin-cerium complex significantly improved cellular uptake and cytotoxic efficacy in comparison to free quercetin, suggesting enhanced photodynamic potential of polyphenols in cancer treatment [16]. In another study, quercetin was reported to sensitize melanoma cells to recombinant human tumor necrosis factor-related apoptosis-inducing ligand [rhTRAIL], a therapeutic agent that has failed clinical translation due to resistance of cancer cells to rhTRAIL-induced apoptosis. Quercetin enhanced rhTRAIL efficacy by upregulating rhTRAIL-binding receptors DR4 and DR5 on the surface of cancer cells, increasing proteasome-mediated degradation of antiapoptotic proteins in cancer cells [17].

Additionally, quercetin has shown promising additive effects when used with other natural compounds. Administration of quercetin with curcumin led to a marked reduction in cell proliferation of several cancer cell lines, including A375 melanoma, A549 lung cancer, HCT116 colorectal cancer, and MCF7 breast cancer [18]. Further investigation realized that this finding was linked to a downregulation of Wnt/beta-catenin, DVL2, beta-catenin, cyclin D1, Cox2, and Axin2 [18]. Similarly, a multi-compound combination of quercetin, curcumin, green tea, cruciferex, and resveratrol was found to inhibit the growth of A2068 melanoma cells [19]. These findings highlight the potential of quercetin-based therapeutic combinations as promising adjuncts in the treatment of melanoma, leading to more effective and multifaceted anticancer strategies.

**Figure 1:**
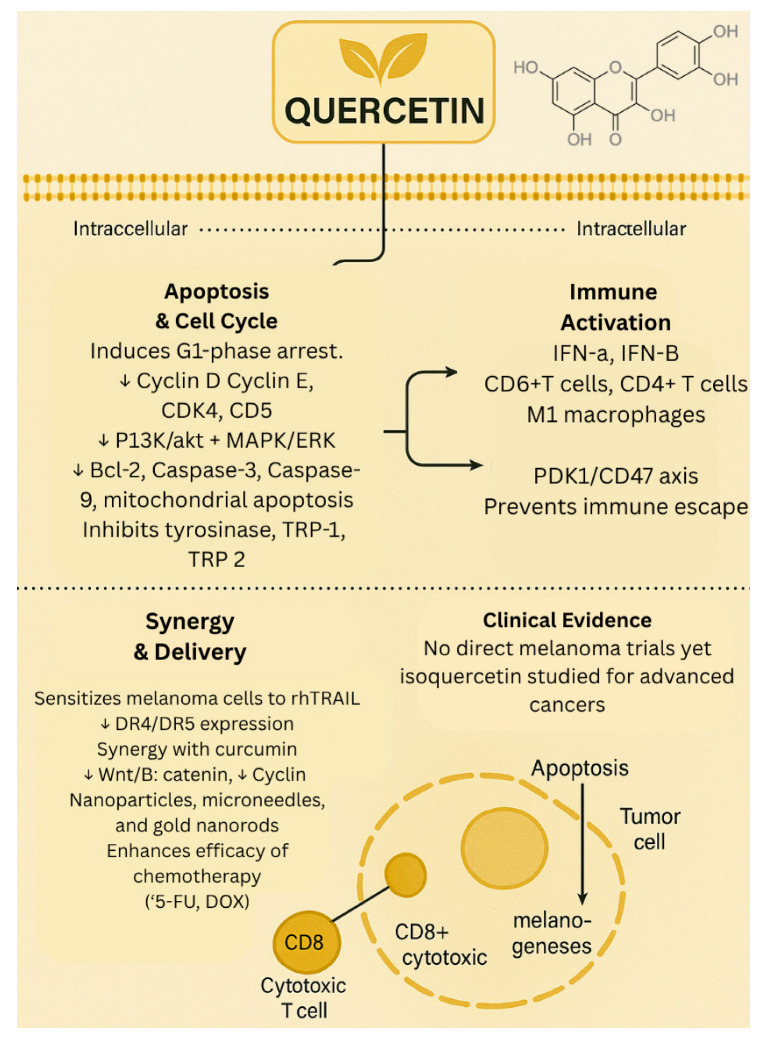
Biological effects of quercetin. ↓—downregulated/decreased expression.

Despite compelling preclinical evidence, clinical data on the efficacy of quercetin for the treatment of melanoma and other cancers remain limited. A phase II/III clinical trial investigated the effects of isoquercetin on hypercoagulability in patients with unresectable or metastatic adenocarcinoma of the pancreas, stage IV colorectal cancer, or stage III/IV non–small cell lung cancer. The study demonstrated that administration of isoquercetin significantly reduced D-dimer plasma concentrations, suggesting improved markers of coagulation in advanced cancer patients [20]. However, no phase II or III trials have evaluated quercetin as a monotherapy or combination therapy for melanoma. This lack of clinical investigation highlights a critical gap in translating quercetin’s promising preclinical anti-cancer effects into clinical application and prompts the need for future clinical trials to evaluate its efficacy and safety in patients with melanoma. The biological effects of quercetin are summarized in Figure 1.

### 1.2. Sulforaphane

Sulforaphane [SFN], a phytochemical predominantly extracted from broccoli sprouts and other Brassica vegetables, has attracted a considerable amount of interest in medical research due to its antiproliferative and cytoprotective properties. This compound is produced secondary to the enzymatic hydrolysis of glucoraphanin, a glucosinolate found in Brassica species [21]. Notably, SFN carries anticancer activity while demonstrating a favorable safety profile with minimal side effects—an uncommon trait among chemotherapeutic agents. Given such properties, SFN has emerged as a promising candidate in cancer treatment and prevention. The unique pharmacological profile of SFN emphasizes the need for continued investigation into mechanisms and therapeutic application in the management of melanoma.

SFN has demonstrated potent pro-apoptotic and anti-proliferative effects in melanoma through multiple signaling pathways. One of the earliest reports by Misiewicz et al. showed that SFN and 2-oxohexyl isothiocyanate induced dose-dependent apoptosis in human melanoma (ME-18) and murine leukemia (L-1210) cells, confirmed by phosphatidylserine externalization and DNA fragmentation [22]. Subsequent studies using B16-F10 melanoma cells further revealed SFN-induced apoptosis marked by caspase-3 and -9 activation, upregulation of Bax and p53, and suppression of anti-apoptotic markers such as Bcl-2, Bid, NF-κB, and caspase-8 [23]. In addition to apoptotic induction, SFN treatment significantly downregulated pro-inflammatory cytokines, including TNF-α, IL-1β, IL-6, IL-12p40, and GM-CSF, in melanoma-bearing mice, indicating immunomodulatory potential [24]. Nuclear translocation of transcription factors such as NF-κB subunits, c-FOS, ATF-2, and CREB-1 was also inhibited, suggesting that SFN acts via a multi-pathway mechanism, further supporting its role in chemoprevention [23]. A broader evaluation of cruciferous-derived isothiocyanates (including SFN) demonstrated that SFN selectively induced cell cycle arrest at the G_2_/M phase in metastatic melanoma cells, accompanied by increased intracellular ROS and glutathione levels [25]. Importantly, melanoma cells showed heightened sensitivity to these effects compared to non-melanoma or normal skin cells, underscoring SFN’s tumor-selective cytotoxicity. Additionally, this highlights the synergistic potential of SFN with other agents.

Oxidative stress plays a critical role in UV-induced melanogenesis and melanoma progression. SFN activates the Keap1–Nrf2–ARE pathway, enhancing transcription of detoxifying and antioxidant enzymes such as NQO1, HO-1, and SOD. In keratinocytes and fibroblasts, SFN-mediated Nrf2 activation was associated with upregulation of IL-6, IL-8, and Bcl-2, promoting cytoprotection against UV-induced oxidative damage [26,27]. Dinkova-Kostova et al. reported that topical application of SFN in SKH-1 hairless mice led to a ~50% reduction in UV-induced tumor incidence, volume, and inflammation. In human subjects, topical SFN extract increased NQO1 activity 2–4.5-fold and reduced UVB-induced erythema by approximately 40%, highlighting its relevance as a chemopreventive agent [26]. Htut et al. further confirmed that SFN produces sustained Nrf2 activation and antioxidant response element (ARE) activity under UV stress conditions [28].

Beyond redox and immune modulation, SFN exhibits significant epigenetic regulatory activity. In B16 and S91 melanoma cells, SFN reduced viability and inhibited HDAC activity. In vivo, SFN-loaded albumin microspheres administered to C57BL/6 mice with B16 tumors resulted in significantly greater tumor volume reduction [~15% more] compared to free SFN and enhanced HDAC inhibition [30% vs. 15%, respectively] [29]. This controlled delivery approach enhanced histone H4 acetylation—a key modification associated with DNA repair and chromatin relaxation—demonstrating SFN’s potential as an epigenetic therapeutic [30]. Collectively, these studies affirm sulforaphane’s capacity to target melanoma through multi-dimensional mechanisms, involving oxidative defense activation, epigenetic remodeling, immunomodulation, and cell death induction.

SFN enhances the efficacy of other anti-cancer compounds, offering synergy through complementary mechanisms. When combined with quercetin, SFN significantly inhibited proliferation and migration of B16F10 melanoma cells more than either compound alone. In vivo, combination therapy suppressed melanoma tumor volume and weight effectively. Mechanistically, this combination suppressed MMP-9 expression and inhibited metastatic potential in melanoma cells [31]. SFN also potentiates the effects of epigenetic therapies. In one study, low-dose SFN paired with 5-aza-2′-deoxycytidine [DAC] enhanced suppression of melanoma cell growth and altered immune gene expression, such as *CCL5* and *IL-33*, more than either agent alone, despite not inducing apoptosis individually [32].

**Figure 2:**
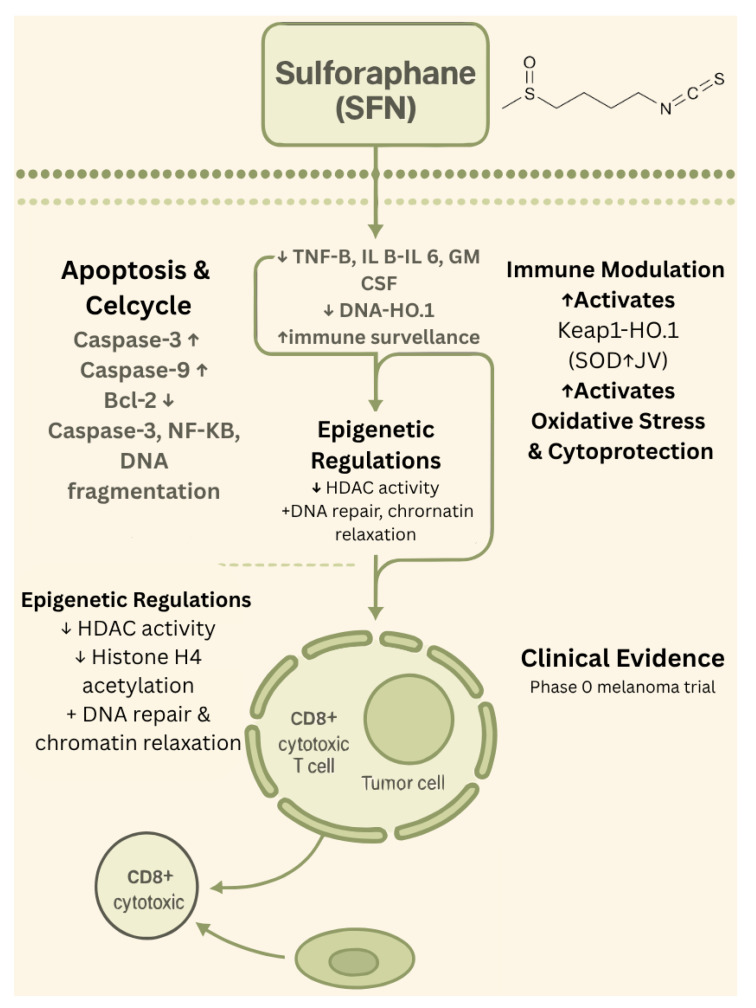
Biological effects of sulforaphane. ↑—upregulated/increased expression. ↓—downregulated/decreased expression.

Only a limited number of early-phase clinical studies have evaluated sulforaphane (SFN) in the context of melanoma. One notable investigation by Tahata et al. (2018) [33] involved a phase 0 randomized controlled trial in 17 melanoma survivors with at least two atypical nevi. Participants received oral broccoli sprout extract standardized to deliver between 50 and 200 µmol of SFN daily over 28 days [33]. The intervention was well-tolerated and demonstrated dose-dependent increases in both plasma and skin SFN levels. Importantly, treatment was associated with a reduction in pro-inflammatory cytokines, including MCP-1, IP-10, and MIP-1β, as well as an increase in decorin expression within nevus tissue, a potential biomarker of tumor suppression. These findings provide early clinical evidence supporting SFN’s bioavailability and biological activity in human skin. Beyond this study, additional early-phase trials in broader oncology populations that included melanoma patients further support SFN’s favorable safety and anti-inflammatory profile; however, melanoma-specific clinical outcomes beyond phase 0 remain largely unreported. The biological effects of sulforaphane are summarized in Figure 2.

### 1.3. Curcumin

Curcumin [1,7-bis[4-hydroxy-3-methoxyphenyl]-1,6-heptadiene-3,5-dione], a lipophilic polyphenol derived from *Curcuma longa* Linn (turmeric) has been used traditionally as a spice, food colorant, and herbal medicine by various cultures for thousands of years [34]. The curcuminoids from turmeric, including curcumin, dimethoxy curcumin, and bisdemethoxycurcumin, are identified as the most bioactive compounds with various health benefits and are highly lipophilic, with increased solubility in various organic solvents and lower solubility in water. Orally administered turmeric compounds have poor bioavailability owing to their rapid metabolism and elimination from systemic circulation, and systemic concentrations of unconjugated curcumin do not exceed low micromolar concentrations in humans despite oral doses ≤12 g [35].

A large body of literature demonstrates the biological effects of curcumin, including cancer-preventive and therapeutic effects [36]. Evidence continues to add new knowledge, focusing on the different molecular and cellular pathways affected by curcumin in lowering the burden of melanoma pathogenesis [37]. A study by Liao et al. showed that curcumin induced apoptosis and caused inhibition of cell proliferation of A375 melanoma cells by regulating cell cycle arrest and cellular redox state. Specifically, the authors identified the role of oxidative stress in contributing to the biological activities of curcumin, which was reversed by the reactive oxygen species inhibitor, N-acetyl cysteine [38]. Similarly, Zhao et al. showed that curcumin induces autophagy and inhibits proliferation and invasion of A 375 and c8161 melanoma cells by downregulating the PI3k/AKT/mTOR signaling pathway [39]. The authors also showed that treatment of BALB/c mice inoculated with A 375 melanoma cells [to establish an explanted in vivo melanoma model] with curcumin for 21 days resulted in tumors that were smaller in size and weight compared to the DMSO-treated control mice [39]. Recently, curcumin was shown to inhibit proliferation, migration, and invasion ability of A 375 and HT144 melanoma cells through miR0222-3p to reduce SOX 10 expression and inactivate the Notch pathway [40]. The authors identified that the miR-222-3p inhibitor reversed the antiproliferative, antimigratory, and anti-invasive effects of curcumin in melanoma cells employed in the study [40].

In a systematic review and meta-analysis conducted by Teng et al. that included preclinical studies focusing on the effects of curcumin in animal models of melanoma, it was concluded that administration of curcumin was relatively safe and has no side effects, and reduced tumor burden by inhibiting growth and cell proliferation [41]. To summarize, various molecular mechanisms have been attributed to the antitumor potential of curcumin, including microRNAs, modulation of critical regulators of apoptotic cell death [caspases, Bax, mTOR, XIAP, Bcl-2 family], factors involved in cell cycle [cyclins, p21, p16], interactions with various growth factors and regulating kinases (TNF-α, IFN-γ, and IFN-α; JAK, MAPK, IKK, PI3K/Akt), transcription factors [NF-kB, STAT-3, p53, p21, p65, and AP-1], and markers involved in chemotaxis, angiogenesis, and metastasis (COX, VEGF, MMP, NOS, and LOX) [37,42].

Curcumin’s potential in overcoming drug resistance in cancer cells has been explored to increase the therapeutic efficacy of existing chemotherapeutic agents. For example, Chiu et al. showed that curcumin induces apoptosis and suppresses tumor proliferation by causing cell cycle arrest, ROS generation, and decreased mitochondrial membrane potential, in addition to downregulating the EGFR signaling pathway in vemurafenib-resistant A 375.S2 melanoma cells [43]. Similarly, curcumin exhibited synergistic anti-cancer effects in malignant melanoma cells [G 361 and SKmEL-2] in spheroid and 2D cultures when used in combination with binimetinib, a MEK1/2 inhibitor, through apoptosis, necroptosis, and ROS production [44]. Recently, curcumin, when used in combination with thymoquinone [a natural compound present in black cumin seeds], synergistically inhibited cell viability, induced apoptosis, and disrupted metabolic and oxidative homeostasis in A 375 melanoma cells [45]. In a different study, the combination of curcumin and disulfiram synergistically inhibited the growth of B16-F-10 mouse melanoma cells and induced apoptotic cell death. The authors also showed that the combination therapy [20 mg/kg curcumin + 60 mg/kg disulfiram] administered intraperitoneally for 15 days reduced tumor weight in an in vivo model employing C57BL/6 mice bearing B16-F10 melanoma cells when compared to the control group or the groups administered curcumin and disulfiram [46].

To increase the bioavailability of curcumin, various strategies/formulations have been developed and tested to evaluate their efficacy compared to the free form. For example, a nano-micellar formulation of curcumin prolonged the survival rates of mice with B16-F10 lung metastatic tumors by increasing the accumulation of activated T cells in the tumor, promoting apoptosis, controlling angiogenesis, and lowering regulatory T cell accumulation [47]. Similarly, oral administration of curcumin loaded into mucoadhesive chitosan-coated polycaprolactone nanoparticles also increased the efficacy of curcumin in preventing melanoma metastasis by inducing apoptosis [48]. Administration of cationic liposomal curcumin in combination with STAT3 siRNA resulted in decreased growth of B16-F10 mouse melanoma cells in culture and tumor reduction in C57BL/6 mice injected with B16-F10 cells when compared with the groups treated with the individual agents [49]. In another study, employing B16-F10 cells, it was shown that curcumin-based nanoformulation enhanced the cellular uptake and cytotoxic effects of the nano-formulated curcumin when compared to group treated with curcumin alone [50].

**Figure 3:**
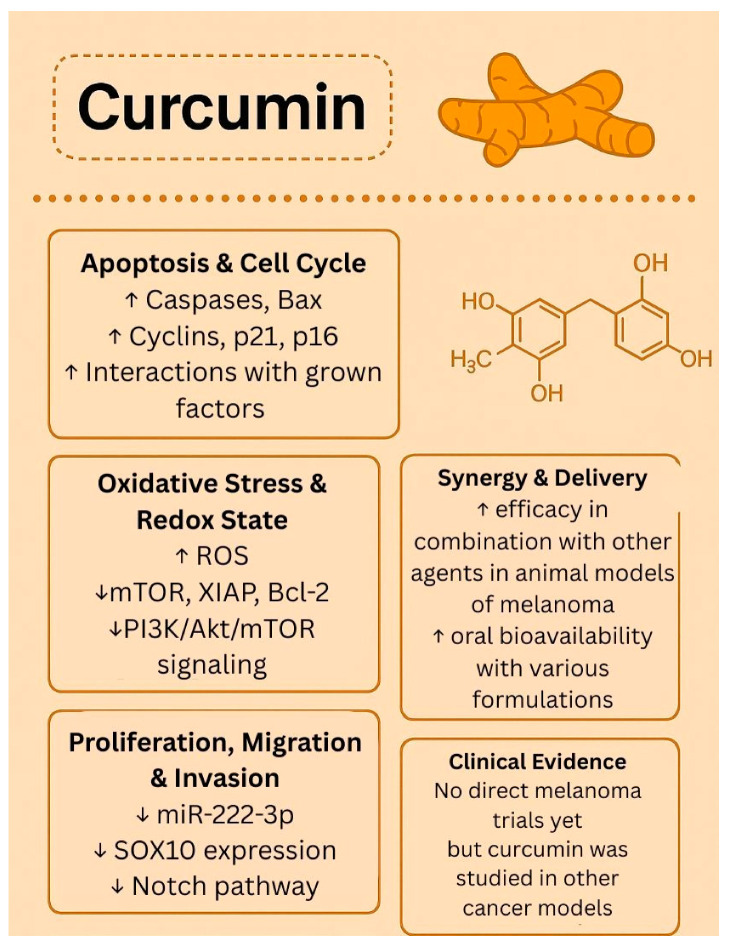
Biological effects of curcumin. ↑—upregulated/increased expression. ↓—downregulated/decreased expression.

While much work has been conducted employing various preclinical models to establish the antitumor potential against melanoma, there are no published human trials testing the therapeutic potential of curcumin for melanoma. A search on ClinicalTrials.gov using the terms “curcumin” and “melanoma” resulted in no studies; however, using the terms “curcumin” and “cancer”, the search yielded 38 completed studies and 14 trials currently recruiting patients. Previously published phase I trials have shown that administration of curcumin is well tolerated at doses up to 12 g but has been identified to have poor bioavailability [51,52]. In a phase IIA clinical trial of curcumin for the prevention of colorectal neoplasia, Carroll et al. showed that curcumin at doses of 2 g and 4 g was well tolerated and decreased the number of aberrant crypt foci, potentially mediated by systemically delivered curcumin conjugates [53]. The biological effects of curcumin are presented in Figure 3.

### 1.4. Epigallocatechin-3-Gallate (EGCG)

EGCG has garnered a lot of attention in cancer research owing to its role in modulating various signaling pathways associated with cancer growth and development. Instead of affecting a single pathway, EGCG is able to interact with many networks at a time, which can regulate cell survival, proliferation, and inflammation. This makes it a highly versatile compound. The PI3K/Akt/mTOR pathway is one of the most studied mechanisms in the field. In melanoma, this pathway promotes rapid cell division and contributes to resistance to apoptosis. Preclinical studies demonstrate that EGCG can inhibit this signaling cascade, leading to a reduction in tumor growth and an increase in programmed cell death [54]. Recent research has also linked EGCG with the induction of autophagy and cell cycle arrest, indicating that it interrupts cancer progression at multiple stages [55].

EGCG also affects immune-related pathways like JAK/STAT. The activation of this pathway is known to contribute to tumor immune evasion. This is achieved by promoting the expression of checkpoint proteins like PD-L1. Studies have shown that EGCG blocks STAT1 activity, which decreases PD-L1 and PD-L2 expression and restores CD8^+^ T-cell function in melanoma models [56]. These findings are relevant because they show signs of EGCG’s potential role in immunotherapies, particularly in patients who do not respond to PD-1/PD-L1 inhibitors. Beyond signaling, EGCG shows genetic effects that also contribute to its anticancer activity. EGCG generates DNA methyltransferases and histone deacetylases, leading to the activation of tumor suppressor genes [57]. This genetic finding demonstrates that EGCG has the capacity to act at both the molecular and genetic levels. While these findings represent a step in the right direction regarding EGCG as a potential candidate, more studies are needed to determine its stability and bioavailability. In its natural form, EGCG is known to rapidly be metabolized, which can affect its impact on cells. Some delivery methods, such as nanoparticle formulations, have been developed to overcome these limitations and have already been proven to improve anticancer activity in animal models [58]. These innovations suggest that EGCG may have the potential to become a new therapy.

Furthermore, apart from its effects on signaling pathways, EGCG plays an important role in regulating the tumor microenvironment and immune responses in melanoma cells. Tumors create immunosuppressive conditions that allow their uncontrollable growth. EGCG’s potential to counter the evasion of immune function by cancer cells is well documented. For example, it has been shown to contribute to the restoration of cytotoxic T-cell activity by decreasing the presence of regulatory T cells (Tregs) and myeloid-derived suppressor cells [MDSCs], both of which weaken antitumor immunity [57]. This sudden change in the tumor strengthens the body’s defense, leading to improved recognition of harm and a more rapid response. EGCG has also been found to be able to lower the expression of checkpoint proteins such as PD-L1 and PD-L2, which are usually used by tumors to avoid their detection [56]. By blocking STAT1 activation, EGCG decreases the checkpoint signals and increases CD8^+^ T-cell function, suggesting that it may work synergistically with immune checkpoint inhibitors [56]. EGCG’s effects on tumor microenvironments are also reflected in its metabolism. Cancer cells often reprogram glucose and lipid metabolism to promote cell growth and suppress immune responses. Administration of EGCG has been shown to reduce glucose uptake and lipid synthesis, ultimately depriving resources essential for tumor growth and development [57]. This also renders tumors more sensitive to immunotherapy and reduces drug resistance. These effects show the potential of EGCG in reshaping the tumor microenvironment and decrease tumor burden. By restoring immune surveillance and disrupting tumor-supportive metabolism, EGCG can strengthen the body’s own defenses.

EGCG’s rapid metabolism is a concern limiting its therapeutic potential against various cancer models [58]. To increase its bioavailability and systemic absorption, several systems have been explored to achieve better results. For example, nanoparticle formulations, liposomes, and polymer-based carriers have shown stronger anticancer activity compared to natural EGCG [59]. These discoveries have not only increased EGCG’s effectiveness but also reduced the required doses to achieve better therapeutic outcomes. Clinical studies, including animal models, have demonstrated that EGCG treatment reduces tumor growth and disrupts tumor-promoting pathways [60]. It has also been shown that EGCG may work synergistically with other treatments, such as immune checkpoint inhibitors or chemotherapy. While clinical trials remain scarce and further studies are required to determine its full effects on different subjects, the effects of EGCG have proven that it may be a promising candidate for cancer models and its therapeutic applications. Its ability to simultaneously interfere with multiple signaling pathways, regulate the tumor microenvironment, and enhance immune responses demonstrate the wide range of mechanisms that make EGCG a candidate for therapeutic treatments.

**Figure 4:**
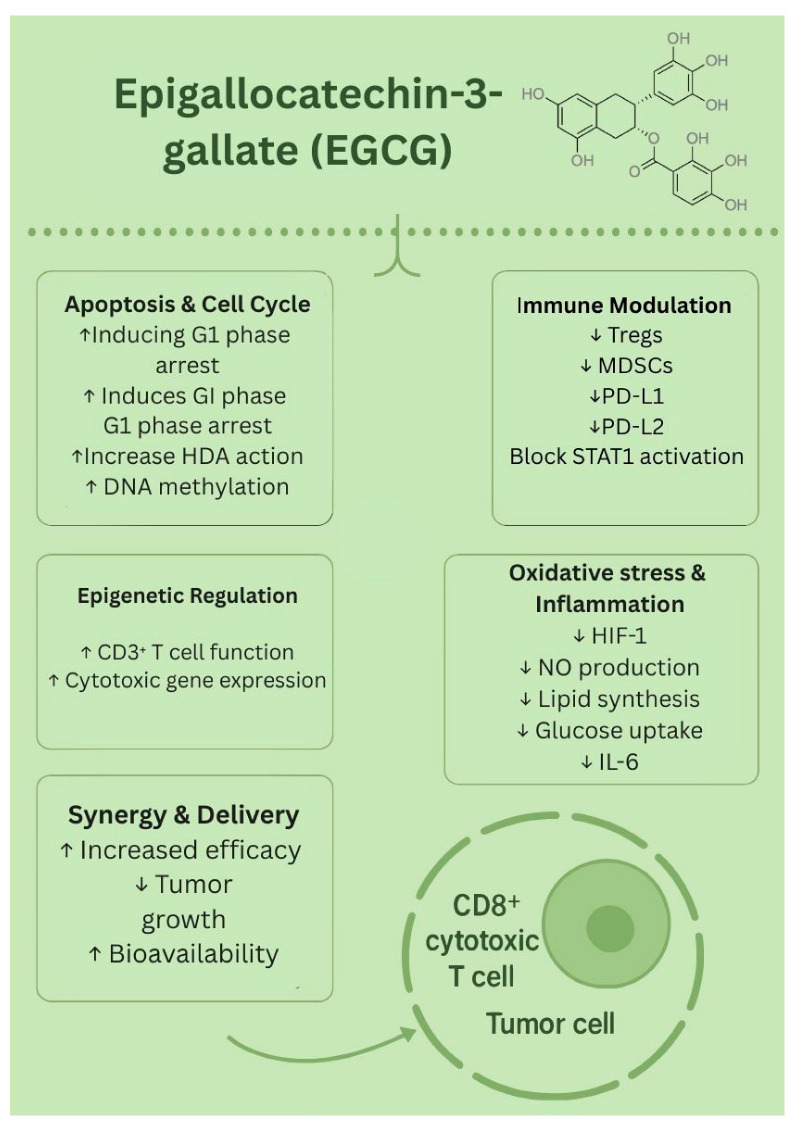
Biological effects of EGCG. ↑—upregulated/increased expression. ↓—downregulated/decreased expression.

Although EGCG faces challenges in its natural form, such as its rapid metabolism and limited stability, new strategies such as nanoparticles, liposomes, and polymer-based carriers have helped overcome these barriers. Preclinical studies have supported EGCG’s role in reducing tumor growth, and evidence suggests the possibility of synergistic effects when combined with chemotherapy or immunotherapy. While clinical trials are still limited, the findings so far indicate that EGCG is a promising natural candidate for future melanoma therapies. The biological effects of EGCG are presented in Figure 4.

### 1.5. Resveratrol

Resveratrol [3,4′,5-trihydroxystilbene] is a naturally occurring polyphenolic compound that has gained significant attention for its potential health benefits. Numerous pre-clinical trials have demonstrated that resveratrol may confer protective and therapeutic effects against diseases such as diabetes, cancer, cardiovascular disorders, and infections through modulating multiple molecular targets and signaling pathways [61]. Perhaps most notable are the antineoplastic effects of resveratrol, which were discovered in 1997 [62].

Resveratrol is a small, lipophilic molecule belonging to the stilbene class of polyphenols [63]. Its molecular formula is C_14_H_12_O_3_, and the structure consists of two phenolic rings connected by a carbon–carbon double bond [64]. The polyphenol exists in both *cis-* and *trans*-conformations, with the *trans*-conformation being more stable and biologically active [65]. Plants synthesize resveratrol to protect themselves from environmental stress, such as microbial infections, ultraviolet light, and physical damage [66,67]. The stilbene is most abundant in the diet from Japanese knotweed (*Polygonum cuspidatum)* and the skin of red grapes [68]. It is particularly concentrated in red wine due to the extended fermentation process that involves grape skins [68]. Smaller amounts are also present in other foods, such as peanuts, pistachios, strawberries, blueberries, and mulberries [68].

Although resveratrol is well-known for its wide range of health benefits including antioxidant, cardioprotective, neuroprotective, anti-inflammatory, antimicrobial, and antidiabetic properties, its clinical use is hindered by its chemical instability and rapid degradation [65,69]. Trans-resveratrol is well absorbed after oral intake but has limited bioavailability due to rapid metabolism and elimination [70]. It undergoes rapid first-pass metabolism in the liver and intestines, converting mostly into less active sulfate and glucuronide forms [68]. As a result, despite good absorption, very little active resveratrol reaches the bloodstream, making it challenging to achieve effective clinical outcomes and determine appropriate dosing [68,71]. Resveratrol demonstrates promising pharmacological activity in inhibiting tumor growth, survival, and metastasis in melanoma. Preclinical studies, including both in vitro cellular assays and in vivo murine models, provide compelling evidence endorsing its efficacy as a chemopreventive and therapeutic agent against melanoma. A growing body of research highlights resveratrol’s ability to suppress melanoma cell proliferation and trigger apoptosis through diverse molecular pathways. One key pathway for this involves the extracellular signal-regulated kinase [ERK1/2] signaling cascade [72,73,74]. For instance, Yu et al. reported that resveratrol treatment reduced the expression of SHC binding and spindle-associated 1 [SHCBP1] in B16 melanoma cells, which caused decreased phosphorylation of ERK1/2 [72]. Given ERK1/2′s role in promoting G1/S cell cycle transition, this inhibition effectively suppressed melanoma cell proliferation [72,75]. Analogously, Zhao H et al. observed that resveratrol inhibited proliferation and induced apoptosis in MV3 and A375 human melanoma cell lines by downregulating the ERK/PKM2/Bcl-2 signaling pathway, which is critical for cell survival and metabolism [73,76]. This downregulation of ERK led to reduced PKM2 activation, decreased expression of the anti-apoptotic protein Bcl-2, and increased expression of the pro-apoptotic protein BAX, collectively promoting apoptosis in melanoma cells [73]. In addition, the study reported upregulation of *p53* [a tumor suppressor gene], contributing further to growth inhibition [73].

Consistent with these findings, a 2018 in vitro study demonstrated that resveratrol triggered cell cycle arrest via the ROS-p38-p53 signaling axis. Expression of p53 and BAX was increased, while Bcl-2 expression levels decreased, thus leading to decreased growth and proliferation of melanoma cells [74]. Moriyama et al. also confirmed that resveratrol inhibited SK-MEL-28 melanoma cell proliferation by halting cell cycle progression at the G1/S phase, specifically by reducing ERK activation and increasing p21 expression [77]. Other studies have shown that resveratrol causes cell cycle arrest by downregulating multiple cyclins and cyclin-dependent kinases (CDKs) essential for cell cycle progression [77,78,79,80]. Yang et al. found that Cyclin D1, a key regulator of G1/S transition, was significantly reduced in A375 and A431 melanoma cells following resveratrol treatment, leading to cell cycle arrest and slowing melanoma cell growth [78,79]. In addition to the downregulation of cyclin D1, several studies have shown that resveratrol treatment also decreases the expression of cyclins A, E, and B1 in melanoma in vitro models, further corroborating the idea that resveratrol prevents melanoma cell proliferation by halting cell cycle progression [77,78,79]. Importantly, Nivelle et al. noted that while resveratrol effectively induced cell cycle arrest in in vitro studies of melanoma cells, it did not adversely affect healthy skin fibroblasts in the same model, highlighting resveratrol’s potential for safe therapeutic use [80]. Further emphasizing resveratrol’s impact on cell cycle regulation, one study found that resveratrol effectively suppressed melanoma cell growth by causing S-phase cell cycle arrest and reduced levels of microphthalmia-associated transcription factor (MITF), a key transcription factor essential for melanoma cell survival. Mokhamatam et al. pointed out that, although resveratrol promotes apoptosis through NF-κB inhibition, its main anti-melanoma effect is driven by the suppression of MITF transcription factor, supporting its promise as a selective therapeutic strategy [81].

Beyond apoptosis and cell cycle control, a review of the literature also identified two studies that reported resveratrol’s ability to reduce telomerase activity [82,83]. Both studies also demonstrated that trans-resveratrol binds selectively to cancer-related G-quadruplex DNA structures to inhibit melanoma cell proliferation [82,83]. G-quadruplexes are four-stranded DNA structures that can form in telomeric regions and suppress telomerase activity, thereby limiting the uncontrolled telomere elongation often seen in cancer cells [84]. Overall, the current literature demonstrates that resveratrol targets a wide range of cellular pathways to suppress melanoma cell growth and promote apoptosis in these cells—including suppression of the ERK signaling pathway, modulation of apoptotic proteins, induction of cell cycle arrest, downregulation of MITF, and inhibition of telomerase activity.

In addition to direct tumor cell cytotoxicity, resveratrol exerts immunomodulatory effects. A 2023 study by Morehead et al. reported that resveratrol increased MHC-I expression in vitro in B16-F10 and A375 melanoma cells, thereby promoting antigen presentation to CD8^+^ cytotoxic T cells [85]. Proteomic analysis in this investigation indicated that this effect may be driven by activation of the stimulator of interferon genes (STING) pathway in melanoma cells [85]. Resveratrol has also been shown to increase the activity of other immune lymphocytes. Lee Y et al. demonstrated that resveratrol significantly enhanced NK cell cytotoxicity and cytokine production, particularly interferon-gamma [IFN-γ], which contributed to suppressed tumor growth in mouse models of melanoma [86]. The same study reported that resveratrol also acted synergistically with interleukin-2(IL-2) to increase these antitumor effects, indicating resveratrol’s promising use for boosting NK cell activity in fighting tumor growth and metastasis in melanoma [86]. In a separate investigation, researchers also found that resveratrol reduced the growth of melanoma cells in vitro and significantly slowed lung metastasis in mice injected with melanoma cells by increasing immune-stimulating factors like CXCL10 and IFN-γ, reducing tumor angiogenesis, and limiting infiltration of immunosuppressive regulatory T cells [Tregs] in the lung [87]. Moreover, another study found that resveratrol reduced the expression of immune-modulating molecules [including IL1α, IL1β, IL6, IL8, TGFβ, MMP2, MMP3, uPA, uPAR, IL6R, IGF-1R, TGFβ-R2, and CXCR4], along with the transcription factor *NF-κB* in senescent fibroblasts [88]. This shift in the tumor microenvironment collectively inhibited melanoma cell proliferation and invasion, suggesting resveratrol’s broader role in promoting immune activation and contributing to melanoma prevention [88]. Together, these studies show that resveratrol boosts immune responses and alters the tumor environment to effectively inhibit melanoma progression and metastasis [85,86,87,88].

Several studies have also demonstrated that resveratrol influences epigenetic and transcriptional regulation in melanoma, contributing to suppressed tumor growth and progression. One key pathway involves the reversal of epigenetic silencing [89]. In melanoma cells, it has been found that the tumor suppressor gene *RUNX3* is often downregulated due to promoter hypermethylation [89]. Resveratrol was observed to significantly reduce this hypermethylation of the *RUNX3* promoter, resulting in an increase in *RUNX3* protein expression, thereby restoring *RUNX3* protein expression both in vitro and in xenograft models, highlighting its role in reactivating tumor suppressor genes [89]. Additionally, other research has shown that resveratrol targets epigenetic regulators such as HDAC1 and DNMT3a, recruiting them to the promoter of focal adhesion kinase [FAK], a protein involved in cell motility and metastasis [90]. By recruiting the epigenetic regulators to the *FAK* promoter, resveratrol increased promoter methylation and reduced *FAK* protein expression, thereby suppressing melanoma cell motility [89]. This suggests a mechanism through which resveratrol may prevent metastasis [90]. Another study also found resveratrol modulated gene expression through microRNA (miRNA) modulation. Specifically, Wu F et al. demonstrated in both in vitro melanoma cells and an in vivo mouse melanoma xenograft model that resveratrol downregulated the oncogenic miR-221 by inhibiting NF-κB signaling [91]. Inhibiting NF-κB signaling, in turn, reactivated expression of *TFG*, a tumor suppressor gene, contributing to resveratrol’s antitumor effects [91].

In addition to its therapeutic effects, resveratrol has shown promise in melanoma prevention. In the reviewed literature, one study developed a resveratrol-based topical gel to deliver resveratrol into the skin for melanoma prevention [92]. The gel demonstrated strong antioxidant properties and cytotoxic effects on melanoma cells [92]. When applied in a mouse model, the gel significantly inhibited tumor growth, indicating its potential utility as a preventive agent against melanoma development [92]. Another study focused on synthesizing novel derivatives inspired by resveratrol by triplicating the core resveratrol structure [93]. These compounds exhibited antioxidant activity, UV protection, and thermal stability, suggesting their possible application as natural, resveratrol-based sun-protective agents to reduce melanoma risk [93].

Collectively, the reviewed literature underscored the broad pharmacological potential of resveratrol in the prevention and treatment of melanoma. Through its ability to modulate key cellular pathways—including apoptosis, cell cycle progression, immune activation, and epigenetic regulation—resveratrol acts through multiple pathways to effectively suppress melanoma growth and progression. Due to its selective activity against melanoma cells and promising outcomes from topical formulations and novel resveratrol derivatives, resveratrol represents a compelling candidate for continued therapeutic development and comprehensive melanoma prevention strategies.

Given resveratrol’s well established anti-melanoma properties, recent studies have examined the synergistic effects of resveratrol when combined with established therapies and found promising results. Emerging literature has also reported the development of novel formulations, such as advanced delivery systems and structural derivatives, that significantly improve the bioavailability and pharmacological activity of resveratrol, thereby augmenting its pharmaceutical potential. In the context of melanoma, resveratrol has shown strong synergistic activity when combined with conventional chemotherapeutic agents, such as 5-fluorouracil (5-FU) [94,95]. Lee et al. utilized a murine model to demonstrate that the co-administration of 5-FU and resveratrol together synergistically decreased melanoma cell proliferation and angiogenesis [96]. Mechanistically, these findings were linked to the downregulation of tumor-promoting molecules, including cyclooxygenase-2 (COX-2), vascular endothelial growth factor [VEGF], and vasodilator-stimulated phosphoprotein (VASP), alongside an upregulation of AMP-activated protein kinase (AMPK) [96]. Thus, while 5-FU may be an effective treatment against melanoma, the literature shows that the combination was more effective than either treatment alone.

Thymidine Kinase/Ganciclovir (TK/GCV) is a suicide gene therapy used in cancer treatment [97]. Chen Y et al. studied the synergistic potential of combining resveratrol with TK/GCV therapy both in vitro and in vivo against B16 melanoma cells [98]. The results indicated that resveratrol improved the efficacy of TK/GCV treatment by upregulating connexin proteins, thus improving gap junction intracellular communication, and, when used in combination, the net effect was increased apoptosis in vitro and a reduction in tumor weight and volume in vivo [98].

Additionally, although several effective chemotherapy treatments are available, melanoma often acquires resistance to these drugs, posing a challenge for long-term efficacy of treatment. However, evidence from multiple studies suggests that resveratrol can help overcome resistance mechanisms in drug-resistant melanoma [94,95,99,100,101]. A 2016 in vitro study by Luo et al. examined the B-RAF (BRAF) inhibitor vemurafenib against melanoma cells resistant to the drug [94]. The researchers utilized cells harboring the BRAF V600E mutation—a common genetic alteration found in nearly 45% of melanomas [102]. Luo et al. concluded that, when added to the treatment regimen, resveratrol inhibited cancer cell proliferation and helped re-sensitize the resistant melanoma cells to vemurafenib treatment [94]. Corre et al. conducted a similar study in 2018 to explore the underlying mechanism by which BRAF V600E/K mutant melanoma cells develop resistance to BRAF inhibitors [95]. Researchers utilized patient-derived xenografts transplanted into in vivo mouse models to discover that the Aryl hydrocarbon Receptor (AhR), a transcription factor, becomes constitutively activated in these resistant melanoma cells, thereby driving treatment resistance [95]. It was further concluded that resveratrol blocks AhR activity, therefore restoring the efficacy of BRAF inhibitors. The combination of resveratrol and BRAF inhibitors was shown to delay tumor progression, highlighting the use of resveratrol as a promising strategy for overcoming drug resistance in melanoma treatment [95]. Resveratrol has also demonstrated synergistic effects when combined with all-trans retinoic acid (ATRA). ATRA is clinically established in the treatment of acute promyelocytic leukemia, but it has also been proposed as a differentiation therapy for targeting stem-like melanoma cells [99]. Stem-like melanoma cells are a subpopulation of tumor cells with stem cell–like properties, including capacity for self-renewal, differentiation, and enhanced resistance [100]. Stem-like melanoma cells are known to develop resistance to chemotherapeutic agents, such as docetaxel [99]. Notably, Kanai et al. reported that resveratrol can increase the effect of ATRA by promoting cellular differentiation, which in turn increases the susceptibility of stem-like melanoma cells to subsequent chemotherapeutic treatment with agents such as docetaxel [99]. Simply put, resveratrol enhances the effect of ATRA by promoting differentiation of resistant stem-like melanoma cells, making the cells more vulnerable to chemotherapy.

In summary, multiple studies have demonstrated that resveratrol not only boosts the effectiveness of conventional melanoma treatments but also helps to overcome common drug resistance challenges. Incorporating resveratrol into combination therapies could therefore offer a powerful approach to improving patient outcomes in melanoma care.

Shifting focus, given that resveratrol’s clinical application is limited by its poor bioavailability and rapid metabolism, researchers have explored strategies to enhance its effectiveness, including formulation with other compounds and incorporation into nanoparticle delivery systems. Extensive research has investigated the therapeutic potential of polyphenols against melanoma, including curcumin, as previously described in this study. However, a recent study developed a solid lipid nanoparticle delivery system designed for the topical co-administration of curcumin and resveratrol [103]. This formulation remained stable for two weeks, inhibited melanoma cell proliferation in vitro, and reduced cell migration, indicating potential to prevent metastasis [103]. Additionally, the formulation demonstrated localized retention, supporting its use as a targeted topical treatment. This research is valuable, as it demonstrates that resveratrol can be formulated into a stable, bioavailable topical treatment with potential efficacy against melanoma [103]. Furthermore, Sim et al. investigated the effects of the flavone compound 7,8-dihydroxyflavone (7,8-DHF) both alone and in combination with resveratrol [104]. They demonstrated that 7,8-DHF inhibited the growth of B16F10 melanoma cells without inducing cytotoxicity by downregulating microphthalmia-associated transcription factor (MITF) and its downstream targets [101]. However, the combination of 7,8-DHF with resveratrol produced enhanced inhibitory effects, highlighting the synergistic potential of these compounds in melanoma treatment [104].

**Figure 5:**
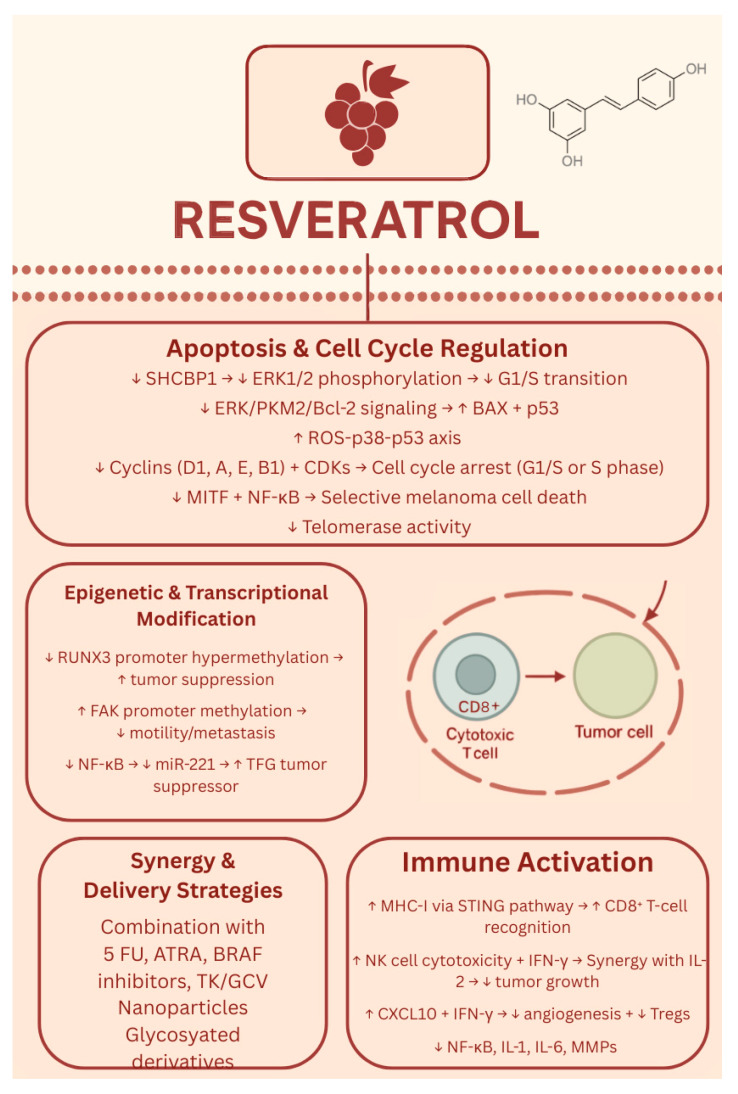
Biological effects of resveratrol. ↑—upregulated/increased expression. ↓—downregulated/decreased expression.

Additionally, to address the limitations associated with resveratrol’s application in melanoma treatment, such as poor solubility, chemical instability, and limited bioavailability, numerous studies have developed nanotechnology-based delivery systems aimed at enhancing its therapeutic efficacy. Across these nine studies, researchers developed a range of resveratrol-based nanoformulations—including polymeric nanoparticles, nanocapsules, transfersomes, cubosomes, mesoporous silica, gold nanoflowers, and organogels—to improve resveratrol’s solubility, stability, and bioavailability [105,106,107,108,109,110,111,112,113]. These delivery systems enabled targeted or controlled drug release, enhanced skin penetration, or pH-responsive behavior, improving resveratrol’s anticancer activity against melanoma in both in vitro and in vivo models. Many studies showed enhanced tumor suppression, apoptosis induction, and reduced metastasis or toxicity compared to free resveratrol [105,106,107,108,109,110,111,112,113]. Overall, nanotechnology-driven delivery strategies present a promising direction for optimizing resveratrol’s therapeutic potential in melanoma treatment. Finally, Moon K et al. developed two glycosylated analogues of resveratrol that exhibited significantly enhanced solubility and stability compared to resveratrol [114]. These derivatives demonstrated improved cellular uptake, with evidence that they are metabolized back into resveratrol intracellularly [106]. This study demonstrates that resveratrol can be modified to have greater bioavailability and biological effectiveness as a drug [114]. The biological effects of resveratrol are shown in Figure 5.

As of August 2025, there have been no clinical trials evaluating the safety or efficacy of resveratrol as a treatment for melanoma. Most of the trials investigated resveratrol’s impact on various types of cancer [colon/colorectal, breast and ovarian, liver, multiple myeloma, lymphoma, and general cancer signaling mechanisms]. Several studies also explored utilizing resveratrol [alone or in combination] to improve hormonal balance, insulin sensitivity, and stress in women with PCOS. Two studies analyzed the safety and bioavailability of resveratrol. One study investigated the incorporation of resveratrol into a novel sunscreen formulation for ultraviolet [UV] protection; however, no results could be identified at the time of review. Overall, among the 18 registered clinical trials involving resveratrol, results and peer-reviewed publications were found for only nine; several trials were either withdrawn, terminated, or lacked publicly available outcome data, and no associated publications could be identified despite comprehensive database searches.

Of note from the clinical trial data reviewed, one trial evaluating a micronized formulation of resveratrol, administered alone or in combination with bortezomib in patients with multiple myeloma, was terminated prematurely due to safety concerns [115]. The published report documented five serious adverse events involving renal toxicity that occurred during resveratrol monotherapy. Although this finding is not directly related to melanoma, it warrants consideration that high supplemental doses of resveratrol may carry a risk of unexpected adverse effects [115].

However, interestingly, other human clinical trials investigating resveratrol supplementation have reported it to be safe [116,117]. One clinical trial investigated the effects of oral resveratrol [0.5 or 1.0 g daily] in 20 colorectal cancer patients prior to surgery, measuring its concentration in colorectal tissues and impact on tumor cell proliferation [116]. Resveratrol and its metabolites were detectable in both normal and cancerous tissues, and a 5% reduction in tumor cell proliferation was observed [116]. The study concluded that resveratrol reaches biologically active levels in the colon and no resveratrol-related adverse events were reported [116]. Furthermore, another clinical trial evaluated the safety and effects of SRT501, a micronized form of resveratrol, in colorectal cancer patients with hepatic metastases [117]. The micronized resveratrol was well tolerated, achieved significantly higher plasma levels than standard resveratrol, was detectable in liver tissue, and increased apoptosis in cancer cells [117]. Overall, current evidence from clinical trials presents conflicting data regarding the safety and efficacy of resveratrol. Additional research is warranted, though future investigations should be approached with caution given the variability in reported outcomes.

### 1.6. Other Phytochemicals

In addition to the well-studied anti-cancer agents discussed in this review, several other phytochemical components have been shown to be effective against various malignancies, but limited evidence is available on their role in the prevention and treatment of melanoma.

**Figure 6:**
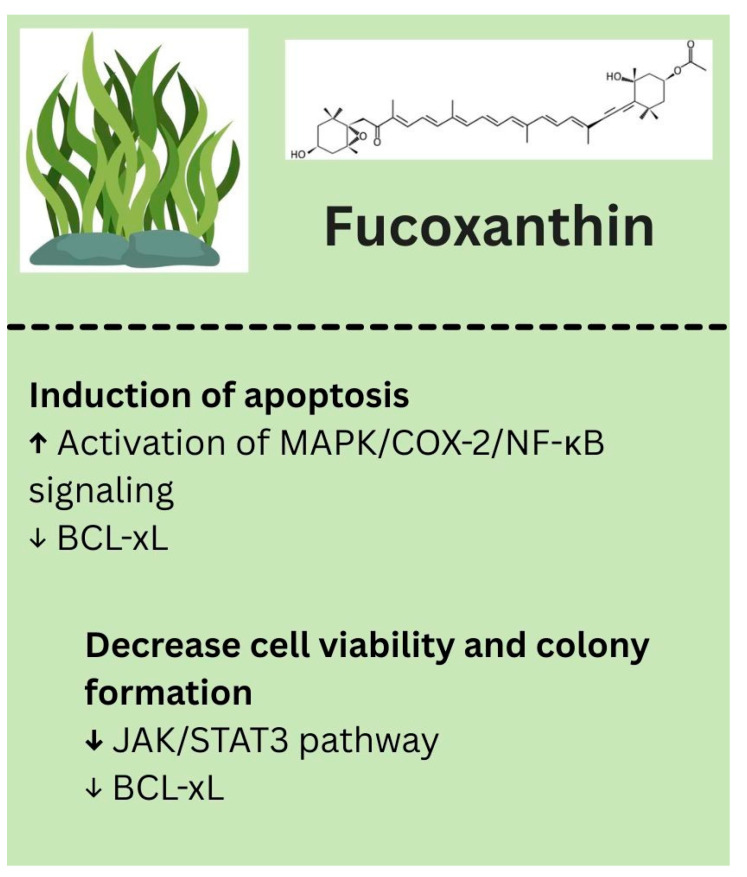
Biological effects of fucoxanthin. ↑—upregulated/increased expression. ↓—downregulated/decreased expression.

Fucoxanthin is an epoxycarotenol pigment found in brown seaweed. Although research on its anti-cancer effects against melanoma is still limited, emerging studies suggest that Fucoxanthin may help prevent melanoma and support skin health by promoting apoptosis, inhibiting malignant cell proliferation, and reducing metastasis. Specifically, Fucoxanthin has been shown to selectively induce apoptosis in melanoma cells by affecting mitochondrial function and activating the MAPK/COX-2/NF-κB signaling pathway [118]. Additionally, it can decrease melanoma cell viability and colony formation by inhibiting the JAK2/STAT3 pathway and downregulating BCL-xL, further triggering apoptotic cell death [119]. The biological properties of fucoxanthin identified in melanoma models are summarized in Figure 6.

**Figure 7:**
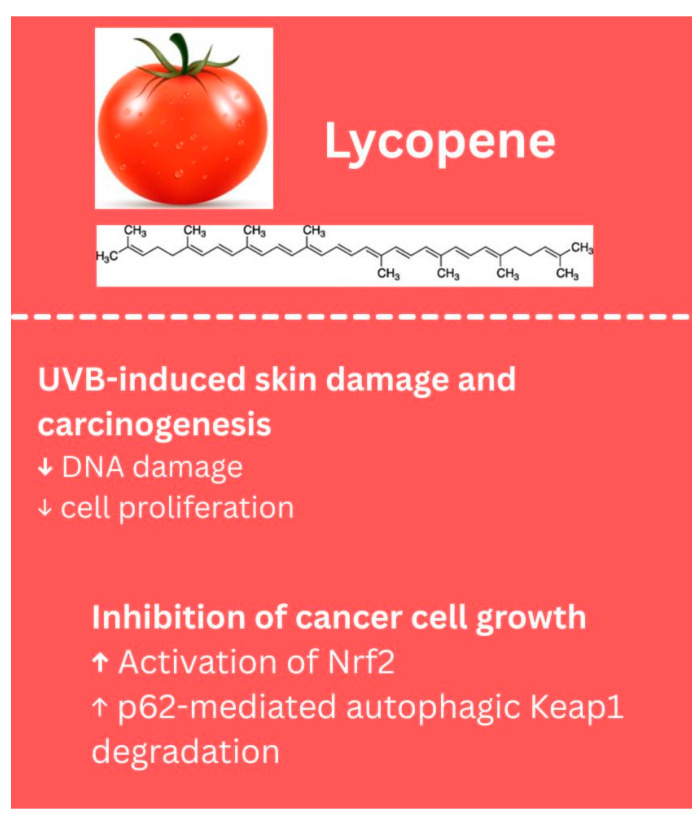
Biological effects of lycopene. ↑—upregulated/increased expression. ↓—downregulated/decreased expression.

Lycopene is another bioactive compound with potential anti-cancer properties, but research specifically examining its effects against melanoma has been limited over the past decade. In an in vivo study, lycopene was shown to inhibit cancer cell growth by activating Nrf2 through p62-mediated autophagic Keap1 degradation, though this evidence was demonstrated in chemically induced skin cancer, rather than melanoma [120]. Additionally, dietary lycopene has been reported to protect against UVB-induced skin damage and carcinogenesis in mice by reducing DNA damage, cell proliferation, and tumor growth; these effects, however, were also not studied in a melanoma-specific context [121]. Literature suggests potential chemopreventive effects in skin cancers, but further studies are needed to evaluate lycopene’s efficacy against melanoma. The biological properties of lycopene identified in melanoma models are summarized in Figure 7.

Altogether, although these lesser-studied phytochemicals show anti-cancer potential, current evidence is incredibly limited and largely preliminary. Future research is needed to investigate their effectiveness against melanoma, their bioavailability in humans against melanoma tumor cells, and their potential synergistic effects when used in combination with other well-studied compounds.

## 2. Conclusions

A wide variety of plant foods consumed across the world are known to have high levels of phytochemicals identified to modulate various biological functions and reduce the burden of developing various chronic diseases. The phytochemicals discussed in the current review have demonstrated health benefits in various preclinical models and clinical trials, including anti-inflammatory, antioxidant, chemopreventive, immunomodulatory, and antitumor properties, among others. In addition, it is their ability to serve as adjuvants and improve the outcomes of chemo- and radiation therapy that makes them an exciting option complementing the traditional therapies. For example, curcumin exhibited synergistic anti-cancer effects in malignant melanoma cells in spheroid and 2D cultures when used in combination with binimetinib, a MEK1/2 inhibitor, through apoptosis, necroptosis, and ROS production. Similarly, resveratrol, when used in combination with 5-FU, synergistically decreased melanoma cell proliferation and angiogenesis in a mouse model. In a different study, treatment of cells harboring the BRAFV600E mutation with resveratrol not only inhibited cellular growth but re-sensitized the resistant melanoma cells to vemurafenib. While these examples serve as a testament to the vast potential of these phytochemicals, there is much to be discovered regarding their therapeutic potential and clinical utility. One major drawback associated with phytochemicals is their lack of bioavailability, which serves as a major deterrent in establishing their role in promoting health benefits. Research strategies have focused on increasing their bioavailability by developing formulations that could potentially increase their availability when compared to the free form. For example, nanomicellar, chitosan-coated polycaprolactone nanoparticles and cationic liposomal formulations have been developed with different phytochemicals. These delivery systems enabled targeted or controlled phytochemical release, enhanced skin penetration, or pH-responsive behavior, improving their anticancer activity against melanoma in both in vitro and in vivo models. Many studies also showed enhanced tumor suppression, apoptosis induction, and reduced metastasis or toxicity compared to the free form. While these preclinical studies provide compelling evidence of the phytochemicals against the development of melanoma, future research should focus on human studies exploring their benefits not only in combating the malignancies but also in improving the quality of patients’ lives. Based on the results compiled, it is reasonable to conclude that the administration of dietary phytochemicals may offer protection against the development of malignant melanoma. Despite these promising cellular and preclinical animal models examined in this article, additional research is essential, focusing on the bioavailability, the development of novel derivatives, and determination of precise concentrations of these bioactive compounds to substantiate their health benefits.

## Data Availability

No new data was created or analyzed in this study. Data sharing is not applicable to this article and the original contributions discussed are included in the references.

## Abbreviations

The following abbreviations are used in this manuscript:

5-FU	5-fluorouracil
7,8-DHF	7,8 dihydroxy flavone
AhR	aryl hydrocarbon receptor
Akt	protein kinase B
AMPK	AMP-activated protein kinase
ARE	antioxidant response element
ATRA	all-trans retinoic acid
Axin2	axis inhibition protein 2
BAX	Bcl-2–associated X protein [pro-apoptotic protein]
Bcl-2 B-cell lymphoma	2 [anti-apoptotic protein]
B-RAF/BRAF V600E/BRAF V600E/K	B-Raf proto-oncogene mutations
CD4	cluster of differentiation 4
CD8	cluster of differentiation 8
CD8^+^	cluster of differentiation 8 [cytotoxic T cell marker]
CDK6	cyclin-dependent kinase 6
CK1	cysteine kinase 1
COX-2	cyclooxygenase-2
CSC	cancer stem cells
CUR	curcumin
CXCL10	C-X-C motif chemokine ligand 10
CXCR4	chemokine receptor type 4
DATS	diallyl trisulfide
DC	destruction complex
DNA	deoxyribonucleic acid
DNMT3a	DNA methyltransferase 3 alpha
DR4	death receptor 4
DR5	death receptor 5
DVL2	disheveled segment polarity protein 2
EGCG	epigallocatechin gallate
EMT	epithelial–mesenchymal transition
ER	estrogen receptor
ERK/ERK1/2	extracellular signal-regulated kinase [1 and 2]
FAK	focal adhesion kinase
GPCR	G-protein coupled receptor
GSK3β	glycogen synthase kinase 3β
HDAC	histone deacetylase
IFN-γ	interferon gamma
IGF-1R	insulin-like growth factor 1 receptor
IL1α, IL1β, IL6, IL8	interleukin 1 alpha, 1 beta, 6, 8
IL-2	interleukin-2
IL6R	interleukin-6 receptor
JAK	Janus kinase
Keap1	Kelch-like ECH-associated protein 1
MAPK/ERK	mitogen-activated protein kinase/extracellular signal-regulated kinase
MDSC	myeloid-derived suppressor cell
MHC-I	major histocompatibility complex class I
miR-221	microRNA-221
MITF	microphthalmia-associated transcription factor
MMP2, MMP3	matrix metalloproteinase 2 and 3
mTOR	mammalian target of rapamycin
NF-κB	nuclear factor kappa-light-chain-enhancer of activated B cells
NK cells	natural killer cells
p21	cyclin-dependent kinase inhibitor p21
p38	p38 mitogen-activated protein kinase
p53	tumor suppressor protein p53
PCOS	polycystic ovary syndrome
PD-L1	programmed death-ligand 1
PD-L2	programmed death-ligand 2
PI3K	phosphoinositide 3-kinase
PI3K/Akt	phosphoinositide 3-kinase/protein kinase B
PKM2	pyruvate kinase M2
rhTRAIL	recombinant human tumor necrosis factor-related apoptosis-inducing ligand
p62	sequestosome-1 (autophagy adaptor protein)
ROS	reactive oxygen species
RUNX3	runt-related transcription factor 3
S phase	synthesis phase of DNA replication
SHCBP1	SHC binding and spindle-associated 1
SRT501	micronized form of resveratrol [drug formulation]
STAT	signal transducer and activator of transcription
STING	stimulator of interferon genes
TCF/LEF	T-cell factor/lymphoid enhancer factor
TFG	tropomyosin receptor kinase fused gene [tumor suppressor gene]
TGFβ/TGFβ-R2	transforming growth factor beta/receptor 2
TK/GCV	thymidine kinase/ganciclovir [suicide gene therapy]
Treg	regulatory T cell
uPA/uPAR	urokinase-type plasminogen activator/receptor
UV	ultraviolet
VASP	vasodilator-stimulated phosphoprotein
VEGF	vascular endothelial growth factor
